# A Case of Pediatric Spitz Melanoma With a *ZEB2::ALK* Fusion

**DOI:** 10.1111/pde.70209

**Published:** 2026-04-08

**Authors:** Nathanael C. Jensen, Shadai Gociman, Sarah D. Cipriano, Casey Mehrhoff

**Affiliations:** ^1^ Department of Dermatology University of Utah Salt Lake City Utah USA; ^2^ Deparment of Pediatric Oncology University of Utah Salt Lake City Utah USA

**Keywords:** cutaneous oncology, genetics, melanoma, pediatric dermatology, Spitz tumor

## Abstract

We present a rare case of Spitz melanoma in a 3‐year‐old male patient with a *ZEB2::ALK* fusion. This *ALK*‐fused tumor exhibited aggressive behavior, recurring after an initial wide local excision and progressing despite neoadjuvant immunotherapy with a PD‐1 inhibitor. Following a second surgical resection and adjuvant radiation therapy, the patient was started on adjuvant targeted therapy with lorlatinib, an *ALK* kinase inhibitor. This report illustrates the role of immunotherapy and targeted therapy in melanoma treatment.

## 1 Introduction

A Spitz tumor is a melanocytic lesion with at least one genetic alteration that typically presents in pediatric patients as a well‐circumscribed, dome‐shaped, red‐pink papule or plaque, or a deeply pigmented brown macule. Spitz tumors account for approximately 1% of all melanocytic nevi in children [1]. The spectrum of Spitz tumors ranges from benign melanocytic neoplasms to Spitz melanoma. The occurrence of melanoma arising in a Spitz tumor is exceedingly rare in children (0.38%–0.5%) [2, 3] and in these retrospective analyses, none of the patients had associated fatal disease. These findings highlight that while most Spitz proliferations in pediatric patients are benign, a small percentage of Spitz tumors can be malignant.

Here, we present a rare case of a recurrent, aggressive pediatric Spitz melanoma in a 3‐year‐old male, with an identified *ALK* fusion.

## 2 Case Report

A 3‐year‐old male with five small congenital melanocytic nevi presented to plastic surgery for evaluation of a separate congenital pink vascular papule near the right ear. The papule was present at birth, had remained pink in color since birth, and initially grew during his first year of life before stabilizing. Eventually, it became increasingly irritated and prone to bleeding with minimal trauma, prompting surgical evaluation. On examination, the posterior right helix had a pedunculated vascular‐appearing papule. Five small additional brown macular congenital melanocytic nevi were noted on the lumbar back and upper thighs with projected adult sizes ranging from 0.9 to 1.5 cm.

**FIGURE 1:**
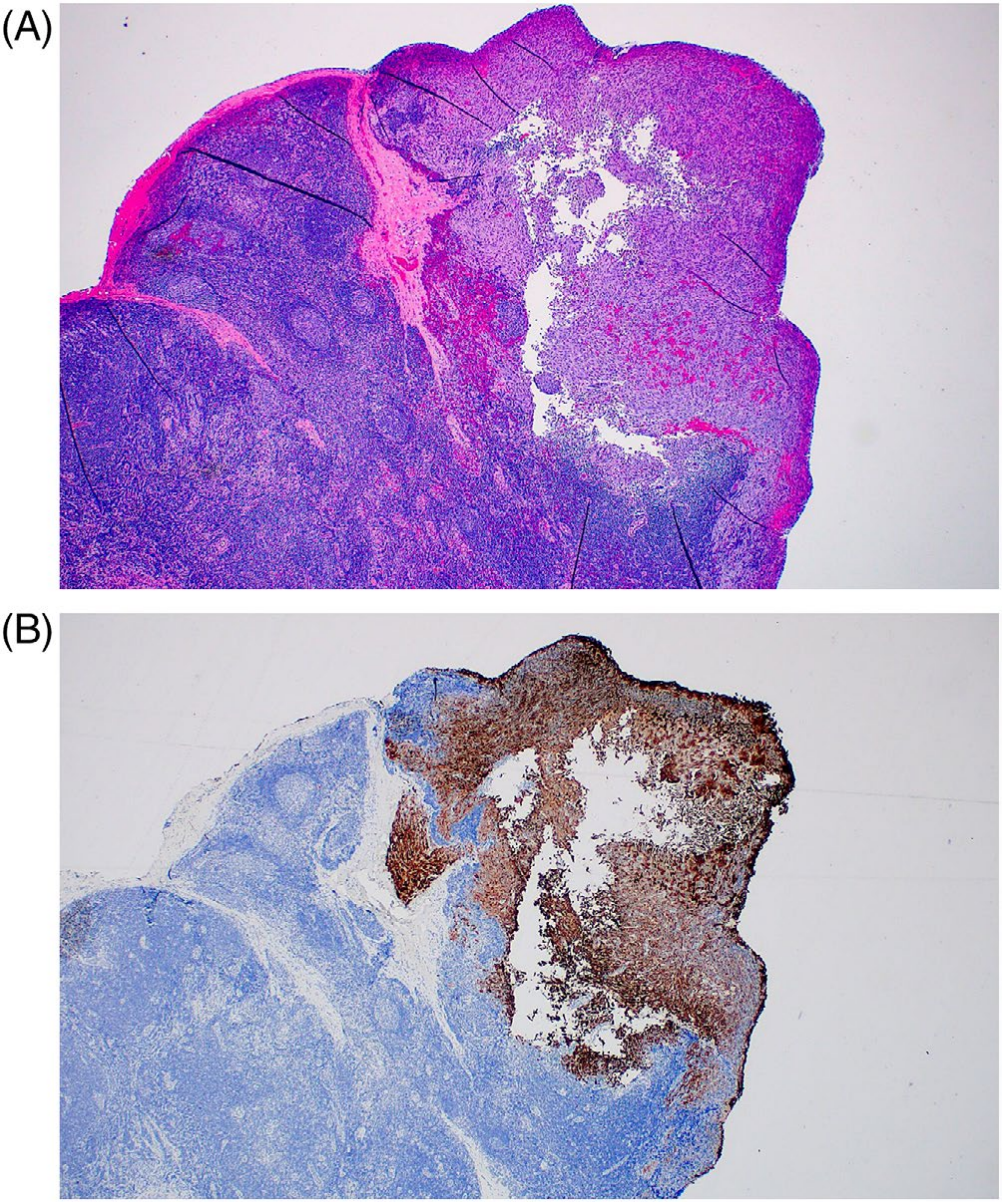
Sentinel lymph node biopsy (H&E, ×40). (A) Encapsulated lymphoid tissue containing a prominent collection of subcapsular pleomorphic spindle cells with focal melanin production. (B) Immunohistochemical stain with Melan A highlighting the cytoplasm of metastatic tumor cells supporting the histologic evaluation.

An elliptical excisional biopsy was performed, with pathology revealing an ulcerated Spitz melanoma with a Breslow depth of 5.7 mm (T4b) and a mitotic count of 7/mm^2^. At this depth there was involvement of the subcutaneous fat. Next‐generation sequencing of the tumor with a 648‐gene panel identified a *ZEB2::ALK* fusion in the tumor cells. The patient underwent a wide local excision, including partial auriculectomy and sentinel lymphadenectomy. Pathology from the excision showed no residual melanoma from the primary lesion; however, 2 of 11 sentinel nodes were positive for melanoma, with tumor foci of 0.2 and 0.3 cm (Figure 1).

**FIGURE 2:**
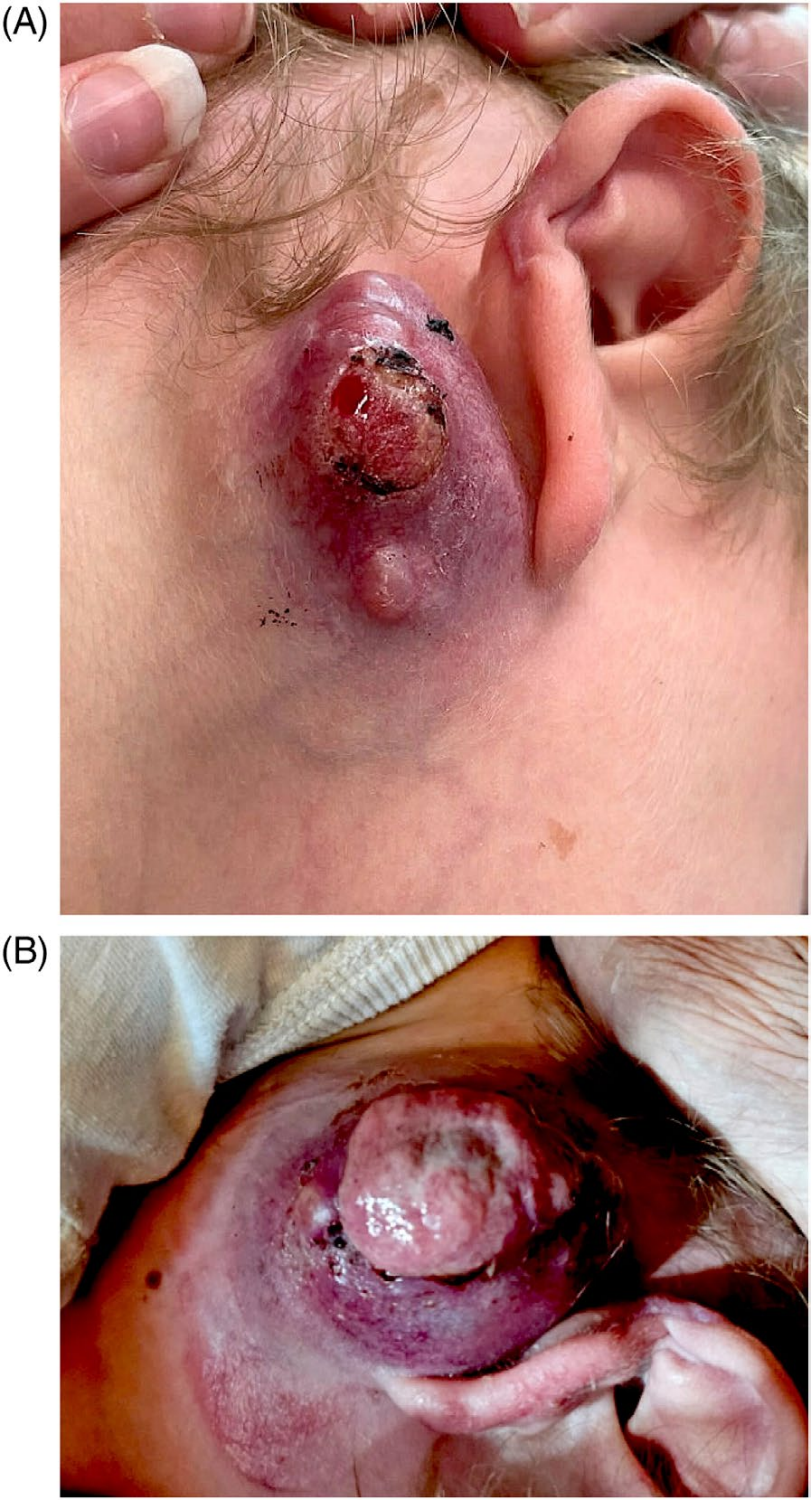
Recurrent Spitz tumor morphology. (A) Growing pink‐violaceous firm tumor nodule 3 months after initial wide local excision. (B) Growth of the tumor nodule after two cycles of neoadjuvant pembrolizumab.

Molecular profiling was negative for *BRAF* or *TERT* mutations. Given the Spitz nature of the melanoma and lack of high‐risk mutations, a decision was made for observation. Three months later, the patient presented with a growing mass behind the right ear at the excision site. Exam revealed an eroded, firm, pink to violaceous nodule (Figure 2A). Ultrasound‐guided core biopsy confirmed recurrent Spitz melanoma, retaining the *ZEB2::ALK* fusion. Brain magnetic resonance imaging and computerized tomography of the chest, abdomen, pelvis, and neck showed no evidence of distant metastases. Based upon the results of SWOG clinical trial 1801, which demonstrated improved event‐free survival in patients 18 years of age and older when three cycles of neoadjuvant PD‐1 therapy were added to the standard of care of surgical resection and adjuvant immunotherapy, the patient was started on neoadjuvant PD‐1 therapy with pembrolizumab [4]. However, after two cycles, the mass continued to enlarge (Figure 2B).

**FIGURE 3:**
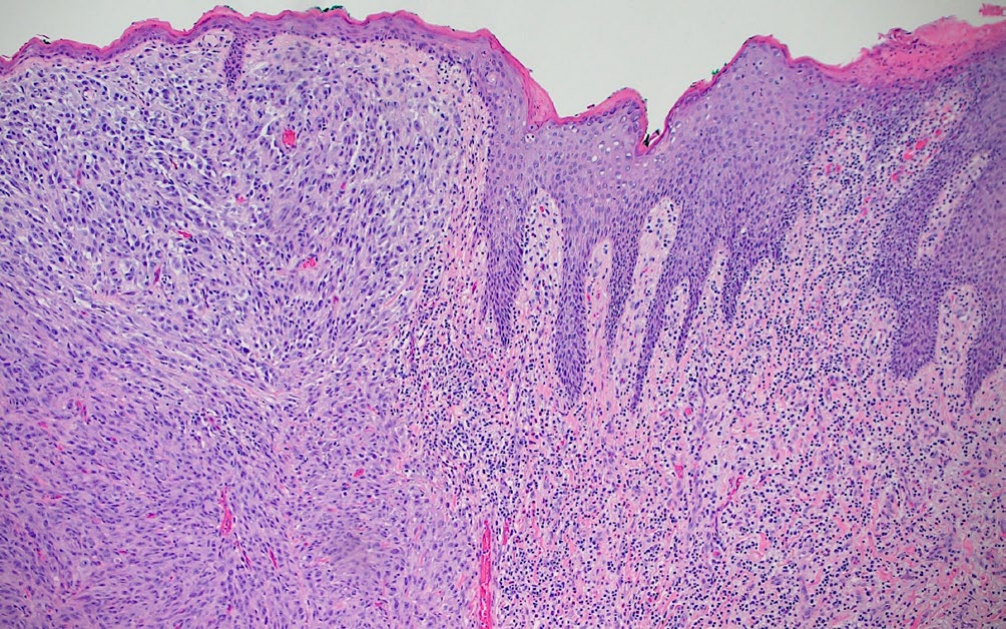
Excisional dermatopathology (H&E, ×100) of recurrent Spitz melanoma. On the left side of the slide there is epidermal atrophy with underlying crowded pleomorphic spindle cells in the dermis with distortion of dermal architecture. The adjacent dermis shows dense lymphocytic inflammation. Note the absence of Kamino bodies and high mitotic index.

Given the progression, neoadjuvant therapy was stopped, and the patient underwent excision with retroauricular and suboccipital lymph node dissection. The surgical procedure included a wide local excision, right parotidectomy, neck dissection, and cervicofacial flap reconstruction. Pathology revealed positive margins for Spitz melanoma, with 7 of 74 lymph nodes positive for melanoma. Excisional histopathology showed crowded pleomorphic spindle cells within the dermis, distortion of the dermal architecture, absence of Kamino bodies, and a high mitotic index (Figure 3).

Due to the aggressive nature of the melanoma with close proximity to facial nerves, surgical morbidity to achieve negative margins was too high, thus requiring further local control with proton radiation. He received 46 Gy to the tumor bed and 55.2 Gy of a planned 60 Gy dose to the areas of positive margins. However, dose‐limiting toxicities prevented completion of the full 60 Gy treatment. Following radiation, the patient was started on adjuvant targeted therapy with the *ALK* inhibitor lorlatinib. Before lorlatinib initiation, there was no clinical or radiologic evidence of residual disease. Lorlatinib was continued for 12 months, with end of therapy imaging demonstrating no evidence of further metastases. Expected clinical side effects were present during lorlatinib therapy, including hyperlipidemia and behavioral changes. The hyperlipidemia was managed with a statin and was discontinued after normalization of his lipids following discontinuation of lorlatinib. His behavioral changes persisted after discontinuation of lorlatinib, ultimately leading to a diagnosis of attention‐deficit/hyperactivity disorder. Unfortunately, 3 months after completion of the 12‐month course of lorlatinib, surveillance CT chest revealed new lung nodules with pathologic confirmation of relapsed disease. Following wedge resection of the pulmonary nodule, lorlatinib was restarted at a higher dose of 100 mg per day. Imaging performed 5 months into the second course of lorlatinib showed no evidence of disease relapse.

## 3 Discussion

Spitz proliferations exist on a spectrum of malignant potential, with expert consensus recommendations available to inform the determination of malignancy [5]. The WHO 2018 guidelines reserve the term “Spitz” lesion for those lesions that have at least one genetic alteration [6]. In contrast, a “spitzoid” lesion is one in which the morphology resembles a Spitz tumor [6]. The classification of these lesions depends on their malignant potential. From lowest malignant potential to greatest, these include benign Spitz nevi, atypical Spitz proliferation, and Spitz melanoma. Histopathologic features and genetic testing can be useful in differentiating benign from malignant lesions. Conventional Spitz tumors are benign, often measuring less than 6 mm, and are rarely ulcerated [5]. In contrast, atypical Spitz tumors have indeterminate malignant potential, are often larger than 6 mm, frequently ulcerated, and can demonstrate asymmetric morphology [5]. Spitz melanomas, which are malignant, are defined as having spindle or large epithelioid cells on histopathology as well as lacking mutations in the *NRAS, BRAF*, and *NF1* genes on sequencing [6]. They also tend to share clinical features of other cutaneous melanomas in pediatric and adolescent patients, such as size > 1 cm, age at diagnosis > 10 years, and ulceration [5]. This case is notable for presenting a Spitz melanoma in a pediatric patient with a lesion on the head and neck, an uncommon presentation.

Dermatopathology evaluation remains the gold standard for distinguishing Spitz tumor subtypes. The histopathological features that may help distinguish among Spitz tumor subtypes include the presence or absence of Kamino bodies, the mitotic rate, and the features of the nucleoli. Kamino bodies, which are eosinophilic hyaline globules, are present in conventional Spitz nevi, but decrease or become absent in atypical lesions and are typically absent in melanoma and *ALK‐*fused lesions, as seen in this case [7]. Mitotic rate is another useful parameter, being typically < 2/mm^2^ in conventional Spitz nevi but > 6/mm^2^ in atypical Spitz tumors and Spitz melanoma. The nucleoli are uniform in conventional nevi, become more prominent in atypical, and are large in melanoma types. Similarly, nucleolar prominence, pleomorphism, and hyperchromatism become increasingly pronounced in malignant subtypes.

The presence of an *ALK* fusion in this case provides new insights into the mutation and fusion patterns of Spitz melanomas. Common mutations in Spitz melanoma include *HRAS* mutations, tyrosine kinase fusions, and serine/threonine kinase [7]. The anaplastic lymphoma kinase (*ALK*) gene, located on chromosome 2p23, encodes a tyrosine kinase receptor that belongs to the insulin receptor family. A study of 140 Spitz tumors found *ALK* fusions in 10% of conventional Spitz nevi, 15% of atypical Spitz tumors, and 3% of Spitz melanoma [8]. Spitzoid melanomas have represented only a small subset of fatal pediatric melanomas. In a multicenter retrospective study including 7 of the 11 centers in the Pediatric Dermatology Research Alliance Pediatric Melanoma Study Consortium, only 3 of 38 fatal melanomas were diagnosed as spitzoid melanoma type [9]. None of these fatal cases were diagnosed in the pre‐pubertal period, with the youngest case being diagnosed at age 13 years. Spitz neoplasms with *ALK* fusions were previously thought to be indolent as neither systemic metastases nor mortality was reported [8]. However, a multi‐institutional report from 2023 presented two newborn boys with large pigmented nodular plaques and satellite nevi with the same *ZEB2::ALK* fusion who developed invasive melanoma before 10 months of age [10]. Both tumors were detected by ultrasound in utero, showed significant nodularity at birth, and rapidly progressed to melanoma within the first year of life. These emerging cases challenge the prior assumption of *ALK*‐fused lesions behaving indolently, demonstrating aggressive behavior when present in both congenital giant nevi [10] and Spitz melanoma. These emerging reports of aggressive pediatric melanomas driven by ZEB2::ALK fusions suggest that this alteration should be included in DNA‐ and RNA‐based next‐generation sequencing panels. Current recommended fusion lists—such as those outlined in Sargen et al.—identify several alternative gene rearrangements but do not include the ZEB2::ALK fusion [5].

Although the five medium congenital melanocytic nevi were not the source of the melanoma in this case, their presence raises the question of whether they may signal an increased risk of de novo melanoma. A 2025 review on melanoma risk in congenital melanocytic nevi supported clinical monitoring, noting that the lifetime risk of melanoma in medium‐sized CMN is approximately 0.3% and that no size‐ or number‐based thresholds currently justify specific screening protocols [11]. In light of these findings, it is more likely that the Spitz melanoma in this case developed de novo and was not related to the five medium congenital melanocytic nevi present at birth.

Lorlatinib, an FDA‐approved *ALK* inhibitor for *ALK*‐positive metastatic nonsmall cell lung cancer, is an investigational agent in pediatric neuroblastoma, but its use in pediatric melanoma is not reported. Thus, clinical outcomes in this case may demonstrate this agent as an effective targeted therapy in these tumors.

The patient's progression on PD‐1 targeted immunotherapy highlights another key consideration. While progression was not entirely unexpected as the patient's tumor mutational burden was 3.7 m/MB, immunotherapy was deemed the best upfront treatment option based on published resources. The MELCAYA study retrospectively analyzed pediatric and adolescent melanoma patients treated with anti‐PD‐1 therapy and reported a 3‐year progression‐free survival of 70.6% and overall survival of 81.1% in the adjuvant setting [12]. The safety profile was consistent with previous studies in adult melanoma patients. In addition to its safety in pediatric patients, immunotherapy has also been shown to be more effective than monotherapy with immune checkpoint inhibitors or combination targeted therapy in metastatic melanoma in adults [13]. This is illustrated by the statement by The Society for Immunotherapy of Cancer which has highlighted the significant improvements in overall survival with the use of immunotherapy, particularly, in combination regimens [14]. However, this case illustrates that neoadjuvant immunotherapy is not universally effective and underscores the importance of close clinical monitoring. There is a paucity of literature regarding the use of immunotherapy on Spitz type melanoma, especially in children. Finally, this case demonstrates the importance of multidisciplinary care in pediatric melanoma management. The patient required coordinated treatment involving plastic surgery, otolaryngology, pediatric oncology, radiation oncology, and dermatology. The patient experienced several side effects while on lorlatinib, including new aggressive mood changes managed by pediatric psychology and hyperlipidemia managed with statin therapy prescribed by cardiology. In healthcare systems where multidisciplinary pediatric melanoma boards are available, we recommend referring patients to these groups to ensure comprehensive, optimized patient care.

## Author Contributions

N.C.J., S.G., S.D.C., and C.M. contributed to the writing and revision of the manuscript. All other authors provided critical input to the case report.

## Consent

Consent for the publication of recognizable patient photographs or other identifiable material was obtained by the authors and included at the time of article submission to the journal stating that all patients gave consent with the understanding that this information may be publicly available.

## Conflicts of Interest

The authors declare no conflicts of interest.

## Data Availability

Data sharing not applicable to this article as no datasets were generated or analysed during the current study.
